# Seed development in *Paeonia ostii* (Paeoniaceae), with particular reference to embryogeny

**DOI:** 10.1186/s12870-021-03373-z

**Published:** 2021-12-18

**Authors:** Keliang Zhang, Weizhang Cao, Jerry M. Baskin, Carol C. Baskin, Jing Sun, Linjun Yao, Jun Tao

**Affiliations:** 1 College of Horticulture and Plant Protection, Yangzhou University, Yangzhou, 225009 China; 2 Joint International Research Laboratory of Agriculture and Agri-Product Safety, The Ministry of Education of China, Yangzhou University, Yangzhou, 225009 China; 3 Department of Biology, University of Kentucky, Lexington, KY 40506 USA; 4 Department of Plant and Soil Sciences, University of Kentucky, Lexington, KY 40546 USA; 5 Department of Plant Engineering, Jiangsu Union Technical Institute, Huai-An, 223200 China

**Keywords:** *Paeonia ostia*, plant embryology, seed anatomy, seed development, storage reserves

## Abstract

**Background:**

Seeds of *Paeonia ostii* have been proposed as a source of raw material for the production of edible oil; however, lack of information about the developmental biology of the seeds hampers our ability to use them. Our aim was to investigate development of the seed coat, endosperm and embryo of *P. ostii* in relation to timing of accumulation of nutrient reserves from pollination to seed maturity. Ovules and developing seeds of *P. ostii* were collected at various stages of development from zygote to maturity. Seed fresh mass, dry mass, germination, moisture, soluble sugars, starch, protein and oil content were determined. Ontogeny of seeds including embryo, endosperm and seed coat were analyzed histologically.

**Results:**

The ovule of *P. ostii* is anatropous, crassinucellate and bitegmic. The zygote begins to divide at about 5 days after pollination (DAP), and the division is not accompanied by cell wall formation. By 25 DAP, the proembryo begins to cellularize. Thereafter, several embryo primordia appear at the surface of the cellularized proembryo, but only one matures. Endosperm development follows the typical nuclear type. The seed coat is derived from the outer integument. During seed development, soluble sugars, starch and crude fat content increased and then decreased, with maximum contents at 60, 80 and 100 DAP, respectively. Protein content was relatively low compared with soluble sugars and crude fat, but it increased throughout seed development.

**Conclusions:**

During seed development in *P. ostii*, the seed coat acts as a temporary storage tissue. Embryo development of *P. ostii* can be divided into two stages: a coenocytic proembryo from zygote (n + n) that degenerates and a somatic embryo from peripheral cells of the proembryo (2n → 2n). This pattern of embryogeny differs from that of all other angiosperms, but it is similar to that of gymnosperms.

**Supplementary Information:**

The online version contains supplementary material available at 10.1186/s12870-021-03373-z.

## Background

The seed development process in angiosperms usually is initiated by double fertilization [1]. That is, one sperm cell fertilizes a haploid egg, and the other sperm cell fertilizes a homodiploid central cell in the ovule, leading to production of a diploid embryo and triploid endosperm [1–3]. The seed coat originates entirely from maternal tissues and is derived from the inner and/or outer layers of the integument [4]. The seed coat provides a pathway for movement of carbon and nitrogen from organ sources to the embryo and endosperm. It also protects the embryo and endosperm, thereby increasing the chance a seed will reach maturity and help establish the next generation [3]. If fertilization fails, the ovule degenerates rapidly, which insures that nutrients invested in the aborted seed are recycled [3]. Many storage compounds accumulate as seeds develop, including carbohydrates (starch), proteins and lipids [4]. These reserves provide about 70% of the calories consumed by humans worldwide. Thus, understanding seed development is of major economic importance and could be valuable information for improvement of seed yield and seed nutritive values [1].

The genus *Paeonia* (Paeoniaceae) is one of the world’s most ancient flowering plant groups [5]. It consists of ca. 35 species of shrubs and perennial herbs that are grown extensively across five disjunct zones of the northern hemisphere: eastern Asia, central Asia, western Himalayas, Mediterranean region and Pacific northwest North America [6]. Members of this genus are widely cultivated for their ornamental and medicinal values [7, 8]. Especially in China, species of tree peony are known as the “king of the flowers”, and they are planted as major garden plants with numerous cultivars and hybrids [7]. Further, many peony species, such as *P. lactiflora*, *P. ostii* and *P. veitchii*, contain chemical compounds with pharmacological activities, such as albiflorin, oxypaeoniflorin, paeoniflorin and paeonol that are unique to this genus [7, 8]. In traditional Chinese medicine, they are prescribed for women’s diseases (dysmenorrhoea or menorrhagia) and for various painful inflammatory conditions such as cholecystitis [8].

In recent years, seeds of tree peonies have been proposed as a source of raw material for edible oil [9], and oilseed peony has become a newly-emergent woody plant oil crop. Seeds of various species and varieties of tree peony contain 24.0–37.8% oil; > 90% of the fatty acids, such as α-linolenic, linoleic and oleic acids, are unsaturated. These fatty acids can provide humans several health benefits, such as lowering blood pressure, inhibiting platelet aggregation during blood clotting and reducing the overall risk for cardiovascular diseases [10, 11].

*Paeonia ostii* is one of the main species proposed for oil seed production. However, tapping of its potential for oil production is hampered by a lack of basic information regarding the developmental biology of the seeds. For example, very little is known about development of the seed coat, endosperm and embryo in relation to timing of accumulation of nutrient reserves, especially fatty acids.

To date, studies of *P. ostii* seeds have focused on seed dormancy and germination [12–14], seed yield, nutrition analysis [15] and fatty acid composition [16, 17]. Although there are some reports dealing with seed development in *P. ostii* [15, 18], they mainly have focused on fatty acid composition and changes in storage reserves of the seeds, and the precise temporal and spatial patterns of formation of the seed coat, endosperm and embryo are lacking. For example, Li et al. [18] divided the development of *P. ostii* seeds into three stages: (i) initial growth (0 to 30 DAP) with low fatty acid accumulation, (ii) rapid growth (31–90 DAP) with accumulation of fatty acid and (iii) maturation (91–100 DAP) with a decrease in fatty acid content. However, fatty acid accumulation alone does not fully characterize the whole seed development process [18]. Han et al. [15] also divided the development of *P. ostii* seeds into three stages: (i) rapid growth (0 to 70 DAP), (ii) slow growth (71–98 DAP) and (iii) maturation (99–112 DAP). However, their first two samples were taken from 14 and 28 DAP, and they did not recognize the slow growth before 20 DAP [15]. In the field, no embryo and endosperm could be seen with the naked eye 40–50 DAP; instead, only the seed coat could be seen. We ask: what happens in the seeds during these stages of growth? For example, during which growth stage(s) is (are) seed coat, embryo and endosperm development completed, and when does accumulation of storage reserves reach its peak?

Our aim was to obtain a detailed picture of seed development of *P. ostii* from pollination to maturity in relation to timing of accumulation of various storage compounds. Thus, for various stages of seed development we collected cytological data on embryo, endosperm and seed coat; soluble sugars, starch, protein and crude fat content; and seed germinability. This information will help identify the stages in seed/endosperm development that are the most critical in terms of oil accumulation and thus potentially assist in development of high oil yielding varieties of *P. ostii*.

Further, the availability and accessibility of draft genome sequences for *Paeonia* species [19] and of a flurry of transcriptomic analyses [20, 21] were designed to define the proteomic changes associated with seed development and the expression patterns of genes involved in the synthesis of fatty acids in *Paeonia* seeds. Nevertheless, because information related to the exact spatial and temporal patterns involved in the formation of the seed coat, embryo and endosperm is lacking, the biological meaning associated with some transcriptomic and proteomic data remains uncertain. This uncertainty usually occurs because tissue samples used in these studies are selected without considering the seed developmental stage, or seed tissues are not separated [21].

## Results

### Morphological changes in seeds during development

**Fig. 1:**
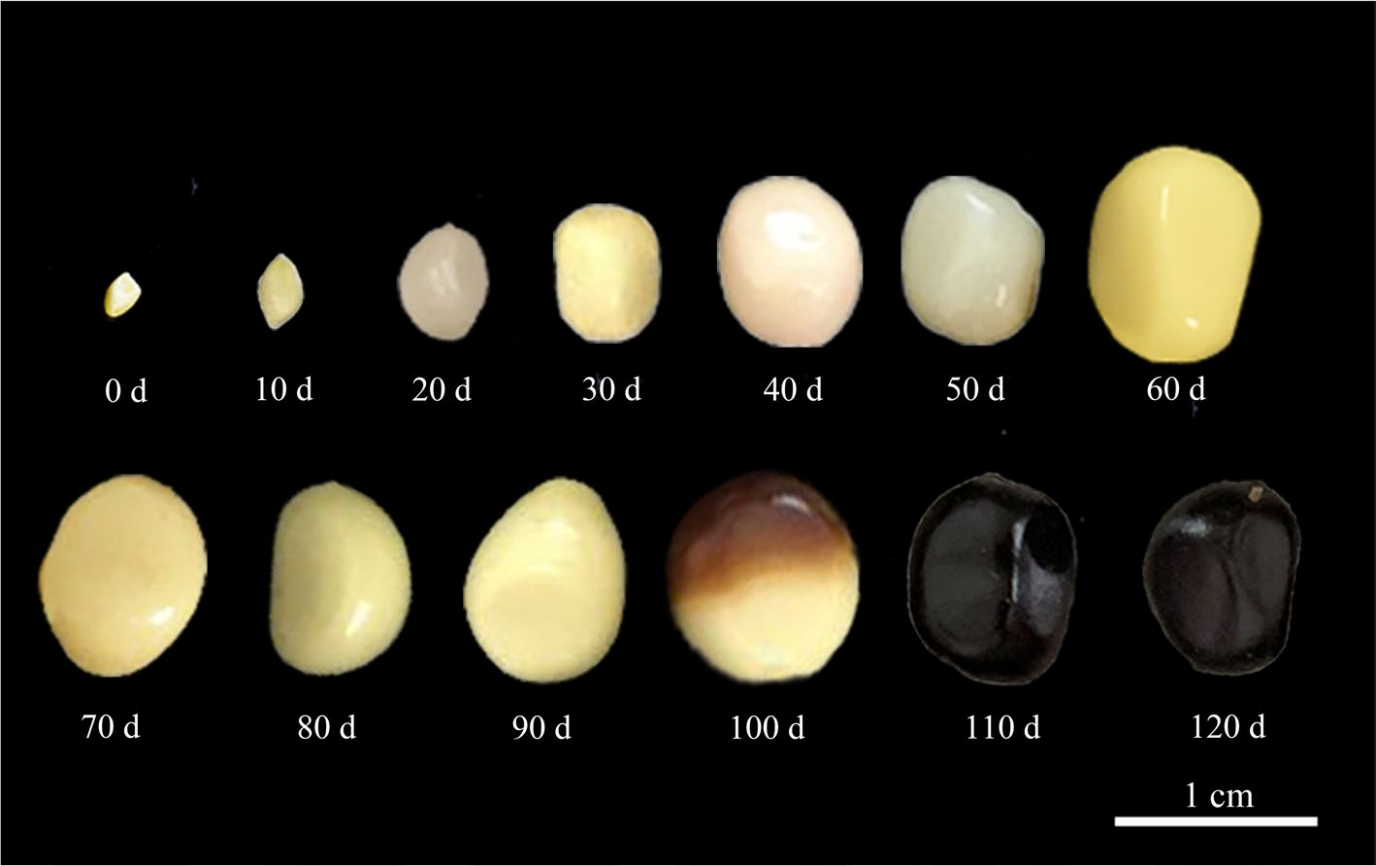
Changes in size and color of seeds (ovule to maturity) of *Paeonia ostii* during development

**Fig. 2:**
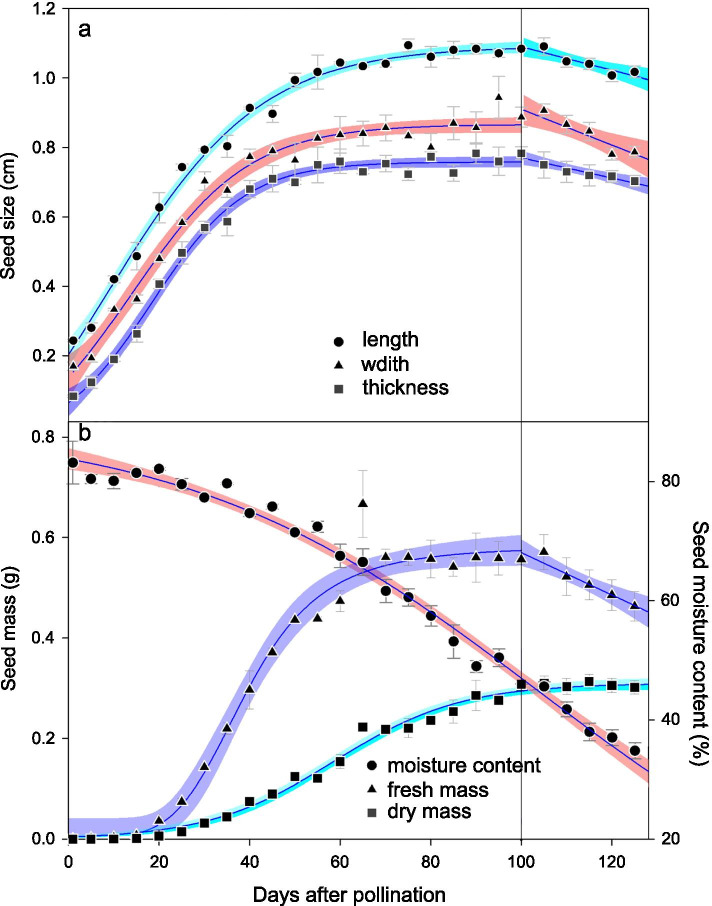
Changes in length, width, thickness, moisture content, fresh mass and dry mass of *Paeonia ostii* seeds during development. (a) Seed length, width and thickness (mean ± s.e.); (b) moisture content, fresh mass and dry mass (mean ± s.e.). Solid lines are regression curves and color bands 95% confidence intervals

In the early stages of development, major changes occurred in the shape of *P. ostii* seeds. At 0 DAP, the ovules are bullet-shaped, but they were rhombus-shaped at 10 DAP. At 20 DAP, seeds were oval, globose or ovoid-globose, and these shapes persisted until 125 DAP, at the time of seed dispersal (Fig. 1). The color of developing seeds gradually darkened over time, with seeds at 0–50 DAP ivory white and those at 60–90 DAP light yellow. At 100 DAP, a dark–brown tinge appeared on the side opposite the hilum in most seeds, and the color gradually spread throughout the seed. The entire seed coat was black by 110 DAP. From 0–65 DAP, seed size gradually increased with seed length > width > thickness, remained constant between 65 and 100 DAP and decreased slightly between100 and 120 DAP (Fig. 2a; Table S1).

### Mass and water content of developing seeds

During seed development, seed dry mass gradually increased, and it peaked at 100 DAP, when the seeds became physiologically mature (Fig. 2b; Table S1). However, from 5–65 DAP fresh seed mass gradually increased; at 65 DAP, fresh mass peaked (0.67 ± 0.07 g). Fresh seed mass remained stable until 100 DAP, when it was 0.55 ± 0.01 g. From 100 to 125 DAP, fresh seed mass decreased (Fig. 2b; Table S1). At 125 DAP, fresh seed mass was 0.46 ± 0.02 g. However, seed water content decreased throughout the period of development (Fig. 2b). Water content at 5 DAP was 83.16 ± 2.07%, and it declined to 41.78 ± 1.27% at 110 DAP and 34.85 ± 1.32% at 125 DAP (Fig. 2b; Table S1).

### Embryo morphology and germinability of developing seeds

**Fig. 3:**
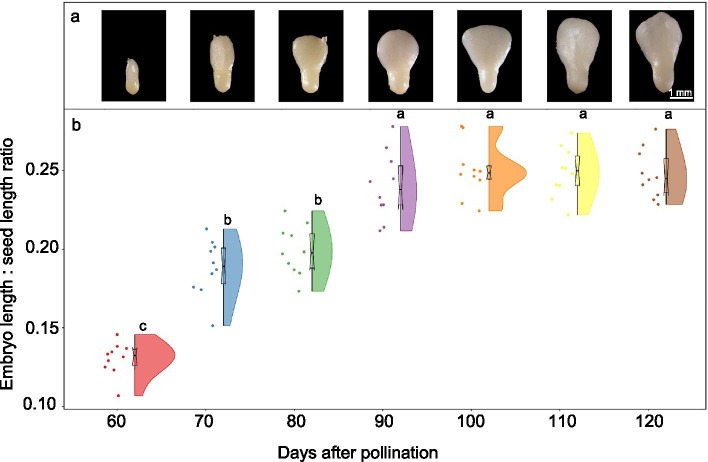
(a) Embryo development (ovule to maturity) morphology and (b) raincloud plot showing embryo length: seed length ratios in developing seeds of *Paeonia ostii.* The raincloud plot combines an illustration of data distribution (the ‘cloud’) with raw point data (the ‘rain’). That is, the colored area (cloud) represents data distribution (the more data points in a specific range, the larger the cloud is for that range). The black boxplot is the interquartile range, middle line the median value and thin line extending from the box the upper (max) and lower (min) adjacent values in the data; the dots (rain) are raw point data. Points are random values assigned to the dots to separate them so that they are not plotted directly on top of each other. Different letters indicate significant differences in embryo length: seed length ratios among different days after pollination (*P* < 0.05)

At 60–65 DAP, the embryo was rod-shaped, and the cotyledons were not fully developed (Fig. 3). At 70 DAP, the cotyledons started to enlarge, and they developed into fan-shaped cotyledons by 80 DAP. In addition, embryo size gradually increased. At 60 DAP, length of the embryo was 1.61 ± 0.08 mm, and at 110 DAP it was 3.29 ± 0.19 mm. In addition, embryo color changed during seed development. At 60 DAP, the cotyledons were translucent and radicles yellow. At 65–85, the whole embryo was yellow and at 90–110 DAP white.

**Fig. 4:**
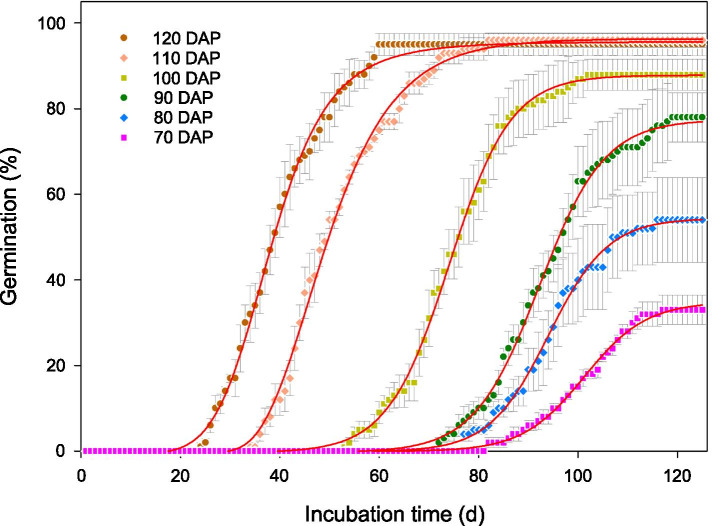
Germination percentages of intact seeds of *Paeonia ostii* at different stages of development (mean ± s.e.)

The developing seeds became germinable at 60–70 DAP. No seeds germinated before 60 DAP, while at 70 DAP 33% did so. Germination increased gradually to the maximum (96 ± 2.6%) by 110 DAP (Fig. 4). Furthermore, as seeds developed germination rate (speed) increased. At 70 DAP, seeds required about 80 days for radicle emergence, while at 120 DAP they required less than 30 days.

### Soluble sugars, starch, protein and crude fat content of developing seeds

**Fig. 5:**
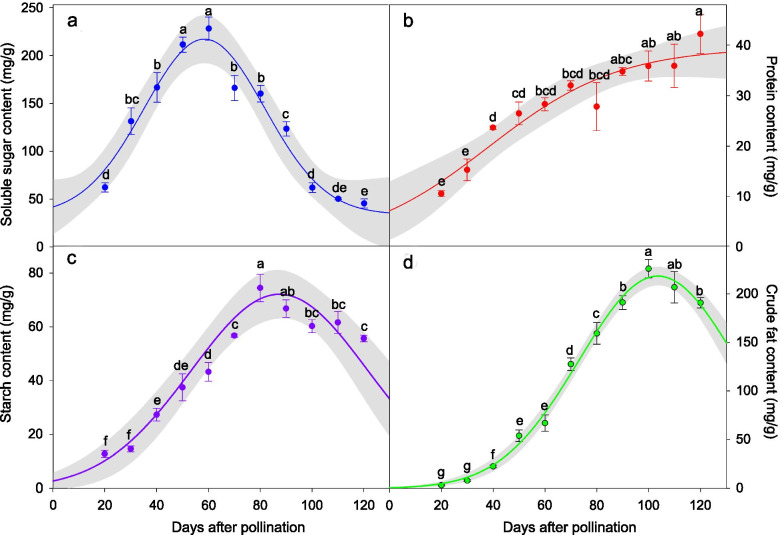
(a) Soluble sugar, (b) protein, (c) starch and (d) crude fat content of developing seeds of *Paeonia ostii* (mean ± s.e.). The solid line is a regression curve and the color band 95% confidence interval. Different letters indicate significant differences among different days after pollination (*P* < 0.05)

Storage reserves changed drastically during seed development (Fig. 5; Table S1). Soluble sugars, starch and crude fat content increased and then decreased, but the date they reached their maximum content differed. Maximum content of soluble sugars was 232 mg/g at 60 DAP, after which it decreased sharply, and by 120 DAP maximum sugar content was only 43 mg/g. Starch content reached a maximum of 73 mg/g at 80 DAP, after that it decreased to 56 mg/g by 120 DAP. Maximum crude fat content was 212 mg/g at 100 DAP, after which it decreased slightly; at 120 DAP, it was 193 mg/g. Protein content was relatively low compared with crude fat, soluble sugars and starch, but it increased throughout seed development; at 120 DAP, it was only 40 mg/g.

### Anatomical changes in seeds during development

**Fig. 6:**
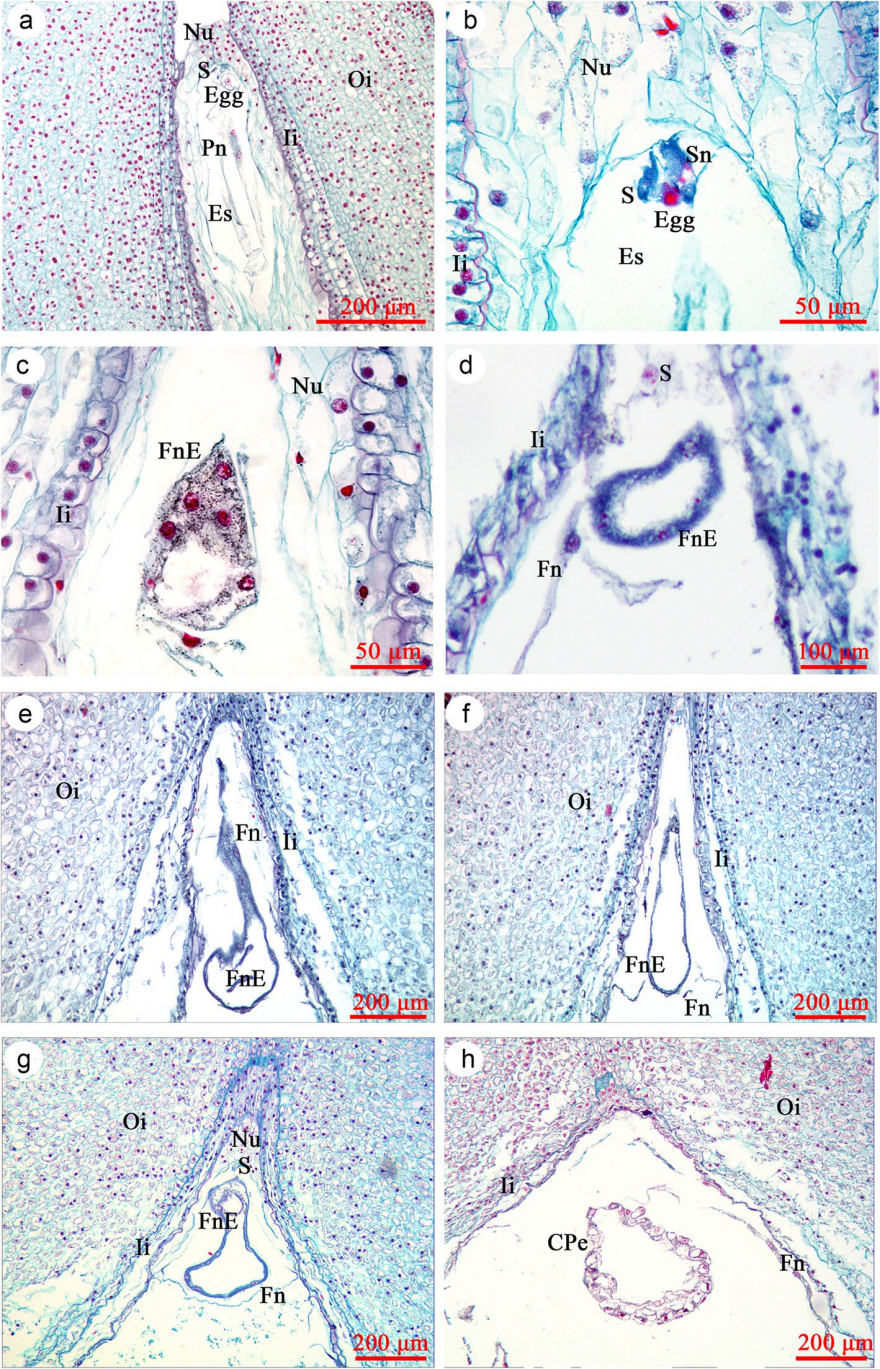
Longitudinal sections of developing seeds of *Paeonia ostii*. (a) 0 DAP (ovule), (b) 3 DAP, (c) 7 DAP, (d) 9 DAP, (e) 13 DAP, (f) 15 DAP, (g) 20 DAP, (h) 25 DAP. CPe, cellularized proembryo; Egg, egg cell; Es, embryo sac; Fn, free nuclear endosperm; FnE, free nuclear proembryo; Ii, inner integument; Nu, nucellus; Oi, outer integument; Pn, polar nucleus; S, synergid cell; Sn, sperm nucleus

The ovule of *P. ostii* is anatropous, crassinucellate and bitegmic (Fig. 6a). The inner integument is composed of 3–4 layers of parenchyma cells, while the outer integument has 14–20 layers of parenchyma cells (Fig. 6a). The mature embryo sac is elongated and located in the center of the ovule, and it is comprised of an egg cell, two synergid cells, three antipodal cells and two polar nuclei (Fig. 6a and b. antipodals not shown). Since the egg and two synergid cells are not in the same plain, they cannot all be shown in the same slide. At 1–5 DAP, the sperm nucleus enters the egg with assistance from one of the synergid cells (Fig. 6b shows two sperm nuclei in synergid).

**Fig. 7:**
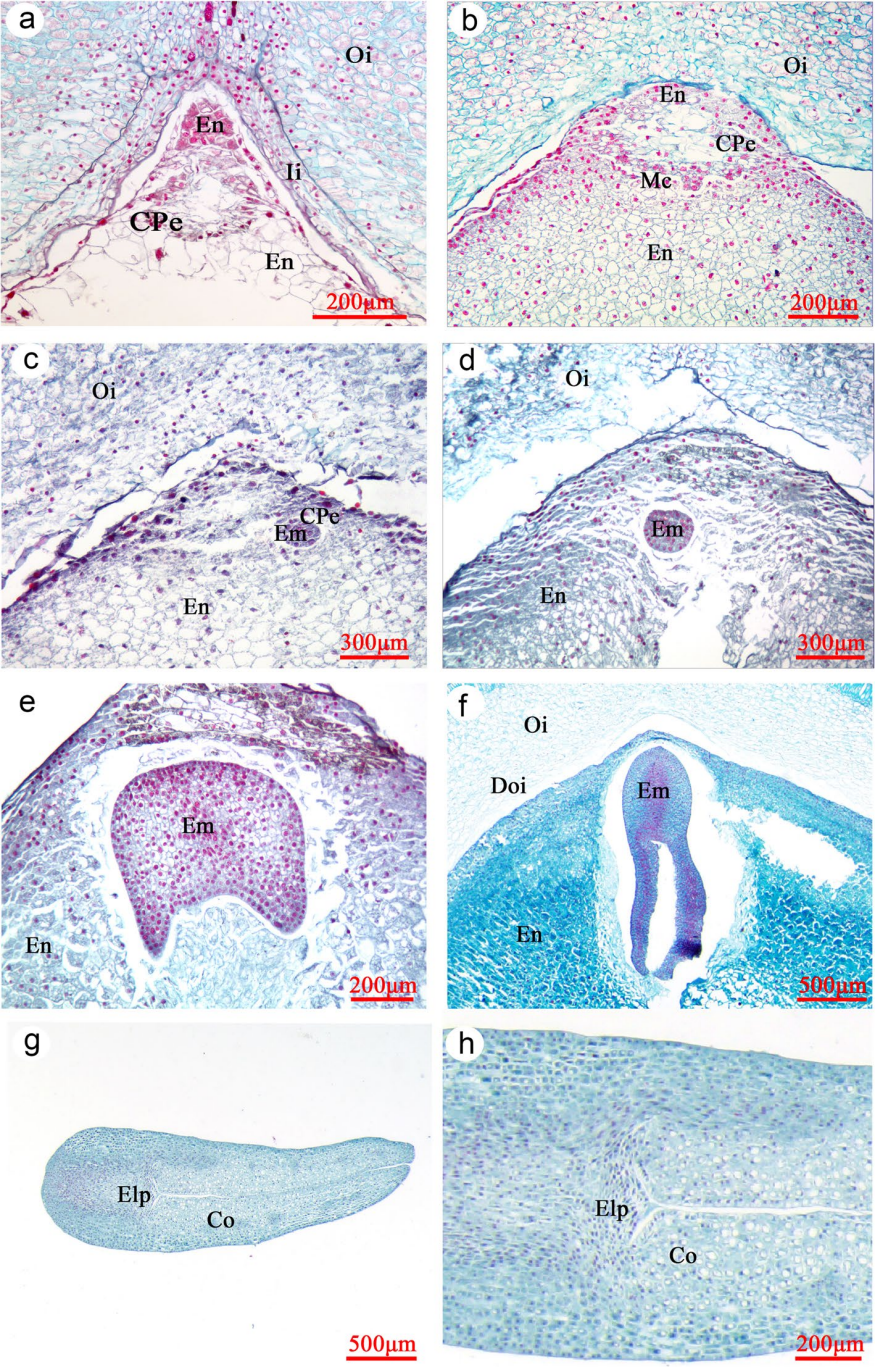
Longitudinal sections of developing seeds of *Paeonia ostii.* (a) 35 DAP, (b) 40 DAP, (c) 45 DAP, (d) 50 DAP, (e) 55 DAP, (f) 60 DAP, (g and h) 90 DAP. Co, cotyledon**;** CPe, cellularized proembryo; Doi, degraded areas in outer integument; Elp, epicotyl leaf primordium; Em, embryo; En, endosperm cell; Ii, inner integument; Mc, meristematic centers of proembryo; Oi, outer integument

After fertilization, the zygote is relocated from the micropyle area to the center of the embryo sac. The zygote begins to divide about 5 DAP, and the division is not accompanied by cell wall formation. Instead, the zygote divides repeatedly and forms a coenocytic proembryo, in which a large vacuole was observed (Fig. 6c). Both synergids are sometimes observed, and one of them may persist until the proembryo reaches a fairly large size (Fig. 6d and g). By 10–15 DAP, these free nuclei are arranged peripherally within the proembryo, and the central vacuole fills the entire proembryo (Fig. 6e and f). By 20 DAP, small vacuoles begin to appear in the cytoplasm at the periphery of the proembryo (Fig. 6g). By 25 DAP, the proembryo begins to cellularize. The free nuclei in the outermost cytoplasm are separated by the periclinal wall, and there are still some free nuclei in the center of the proembryo (Fig. 6h). By 35 DAP, almost all of the free nuclei have formed a cell wall, the central vacuole has shrunk and the proembryo is a solid mass of cells (Fig. 7a). By 40 DAP, certain peripheral cells have divided, the margins of the proembryo are undulated at the surface and meristematic centers have formed, each of which has the potential to become an embryo primordium (Fig. 7b). At 45 DAP, only one embryonic protuberance has continued growing, and it forms a globular embryo; the other coenocytic structures have collapsed (Fig. 7c). Size of the proembryo gradually increases, becomes compressed and gradually disintegrates at 50 DAP (Fig. 7d).

By 55 DAP, two cotyledon primordia can be seen away from the chalazal side of the seed, and the embryo has become heart-shaped (Fig. 7e). Thereafter, the cotyledons differentiate rapidly, and the whole embryo undergoes extensive elongation and matures. By DAP 60, the embryo can be seen with the naked eye (Figs. 3 and 7f). Thereafter, growth of the cotyledons is responsible for much of its increase in length, rather than growth of the axis. However, the leaf primordium does not appear until 90 DAP, when a bulge was observed between the two cotyledons (Fig. 7g and h). Thereafter, embryo morphogenesis is completed, and no further morphological change occurs.

**Fig. 8:**
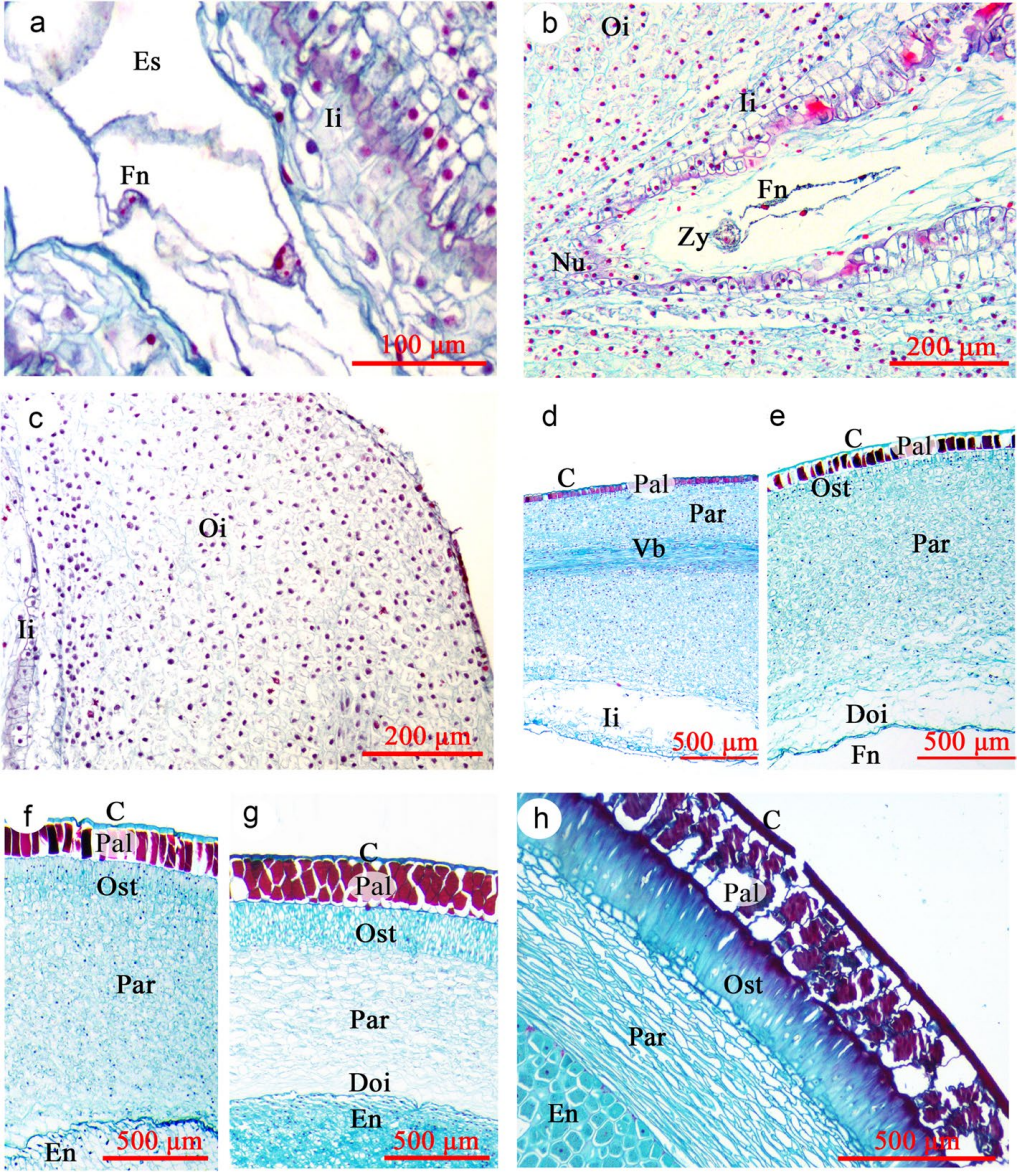
Longitudinal sections of developing seeds of *Paeonia ostii.* (a) 3 DAP, (b) 5 DAP, (c) 9 DAP, (d) 20 DAP, (e) 30 DAP, (f) 35 DAP, (g) 45 DAP, (h) 120 DAP. C, cuticle; Doi, degraded areas in outer Integument; En, endosperm cell; Es, Embryo sac; Fn, Free nuclear endosperm; Ii, inner integument; Nu, Nucellus; Oi, outer integument; Ost, osteosclerid layers; Pal, palisade cell layers; Par, parenchyma cell layers; Vb, vascular bundle; Zy, zygote

Development of the *P. ostii* endosperm follows a typical nuclear type, whereby the primary endosperm nucleus divides without formation of a cell wall and ultimately produces a peripheral multinuclear endosperm tissue. Endosperm development occurs earlier than that of the embryo (Fig. 8a and b). The endosperm has 8 to 16 nuclei before the zygote divides. As the free nuclei of the endosperm repeatedly divide and proliferate, they are gradually distributed at the periphery of inner integument (Fig. 6d, f and h). Cellarization of the free nuclei endosperm occurs between 30–35 DAP, which is later than that of the proembyo. Cell wall formation of the free nuclear endosperm is rapid, and it begins adjacent to the proembryo and spreads toward the chalazal end of the seed (Fig. 7a). During cellularization, the endosperm gradually changed from liquid to colloidal state. The inner integument is gradually degraded and absorbed by the endosperm cells. By 40 DAP, all endosperm free nuclei form a cell wall (Fig. 7b), and no inner integument can be observed. Further, the inner layer of the outer integument gradually degrades, and a clear imprint of the degraded area of the outer integument was observed (Fig. 8g). From 50 DAP, inclusions in the endosperm cells gradually increased (Fig. 7d-f). By 65 DAP, the endosperm was solid, and the cells were full of inclusions (nutrition). Thereafter, the endosperm tissue gradually hardens and reaches a mature state.

The seed coat of *P. ostii* develops from the outer integument. During 1–10 DAP, the integument cells progressively grew with no obvious differentiation (Fig. 8c), while those of the outer integument divided periclinally, with thickest areas having more than 50 layers. Thus, the thickness of the seed coat increased. Thereafter, the outer epidermis grew periclinally, and a cuticle layer was observed above it. By 20 DAP, the epidermis was stained red by safranin, indicating that the cell wall had become lignified. Further, a palisade cell layer of macrosclerid cells had formed (Fig. 8d). A vascular bundle often was observed at the middle of the outer integument during development (Fig. 8d). As seeds developed, the inner integument gradually was absorbed by the endosperm (Fig. 7b). By 30 DAP, the layer beneath the palisade cell layer had elongated, forming the osteosclerid layer (Fig. 8e). As the endosperm cellularized, thickness of the seed coat gradually decreased, with the maximum thickness observed at 35 DAP. Prior to 45 DAP, when the endosperm changed to the colloidal state, the principal tissue of the developing seed was the seed coat (inner integument), which contributed most of the seed mass. Thereafter, the palisade and osteosclerid cell layers continued to elongate, and the seed coat could be divided into four distinct parts, which were (from outside to inside): cuticle and palisade, osteosclerid and parenchyma cell layers (Fig. 8f and g). Before seed dispersal, the parenchyma cells have contracted due to the compression on them by the developing endosperm (Fig. 8h).

## Discussion

Cultivation of oilseed peony as a woody edible oil crop is currently in the early stages of development, and heretofore the precise temporal and spatial patterns of formation of the seed coat, endosperm and embryo were lacking. In this study, we present the results of a detailed account of the morphology/anatomy of seed development, accumulation of storage reserves and germinability of *P. ostii* seeds at various stages from fertilization to seed maturity.

### Growth and development of the *P. ostii* seeds

Mature seeds of *P. ostii* consist of an embryo, endosperm and seed coat, with the endosperm occupying most of the volume inside the mature seed. Development of the embryo of *P. ostii* differs from that of all other angiosperms, but it is similar to that of gymnosperms (see below). Endosperm development of *P. ostii* follows the typical nuclear type (see Sreenivasulu and Wobus [22] for types of endosperm development). Cellarization of the free endosperm nuclei occurs later than that of the proembyo, which differs from *P. californica* in which the nuclei are cellarized at the same time [23]. The seed coat of *P. ostii* is derived from the outer integument, and during development its color changes from yellow to black.

Development of most orthodox (desiccation tolerant) seeds can be divided into three phrases: (I) early embryogenesis, (II) cell expansion and accumulation of stored reserves and (III) maturation drying. The duration of each of the major phases of development varies from several days to several months, depending on species and environmental conditions [4]. For instance, *Geranium carolinianum* seeds reached physiological maturity 14 DAP [24], *Lotus ornithopodioides* 54 DAP [25], *Cypripedium formosanum* 120 DAP [26], *Ginkgo biloba* 190 DAP [27] and *Albizia lebbeck* 230 DAP [28]. The time span of seed development in *P. ostii* from pollination to maturity was 125 d. Seeds reached physiologically mature by 100 DAP, during which time dry matter content reached its maximum. The whole developmental process in *P. ostii* is longer than that reported for other peony species. For example, seeds of *Paeonia rockii* required 100 DAP from pollination to seed maturity, while those of *P. lactiflora* ‘Hangshao’ required 90 DAP, with seeds reaching physiologically mature by 70 DAP [29].

Early embryogenesis (phrase I), cell expansion and accumulation of stored reserves (phrase II) and maturation drying (phrase III) of *P. ostii* occurred 1–60, 21–100, and 101–125 DAP, respectively. These results are quite different from those of other well documented orthodox seeds, in which these three phrases are discrete and continuous. For example, in *Arabidopsis thaliana* and *Glycine max* [30] the embryo is globular, then heart-shaped and forms a ‘linear cotyledon-shaped’ embryo during phrase I, and the embryo axis and cotyledons are formed (differentiated) at the end of early embryogenesis [30], i.e., phrase I. However, in *P. ostii*, seed dry mass increased quickly from 20 DAP, indicating the start of synthesis and deposition of stored reserves, i.e. start of phrase II. However, at 20 DAP the embryo is not well differentiated and still in a coenocytic proembryo state; a ‘linear cotyledon-shaped’ embryo was not observed before 60 DAP. This indicates that the first two phases of embryo development overlapped. In phrase III, seed dry mass reached its maximum and remained constant, while fresh mass decreased, which may mean that seeds underwent an acute loss of water because of a loss of vascular supply to the seed [4]. However, we did not find an acute loss of water in seeds of *P. ostii.* We speculate that this may have been due to the accumulation of storage reserves in phrase II that consumed a large amount of water and thus masked the water loss. A displacement of water by insoluble reserves from cytoplasm is common during accumulation of storage reserves in the development of orthodox seeds [4].

### Embryogenesis

Division of the zygote of *P. ostii* is not accompanied by cell wall formation, and according to available references a coenocytic proembryo is formed. These results are consistent with those of Yakovlev and Yoffe [31] for *P. anomala*, *P. daurica* subsp. *wittmanniana* (as *P. wittmanniana*) and *P. suffruticosa* (*as P. moutan*), and they agree with the report of Cave et al. [23] on *P. californica* and *P. brownii* and of Mu and Wang [32] on *P. lactiflora*. However, our results do not agree with those of Murgai [33] that the first division of the zygote nucleus is accompanied by cell wall formation and that the suspensor is coenocytic [33]. Prior to division of the zygote nucleus and even following the first several divisions, it appears that one synergid cell is pressed against the developing zygote. Thus, the two-cell proembryo of Murgai [33] should be the surviving synergid cell that forms a unit with the zygote. In addition, we did not find an apical cell attached to the coenocytic proembryo.

An extensive free-nuclear stage at the beginning of embryogeny is common among gymnosperms. Moreover, there is a trend in reduction of free nuclei in evolutionarily advanced gymnosperms [34, 35]. For example, some species of cycads can have more than 2^10^ (1024) free nuclei in the early stages of embryo development. In *Ginkgo biloba* (Ginkgoales), several hundred free nuclei are uniformly distributed in the embryo sac [36], while in Coniferales such as *Picea* and *Pinus*, the number of free nuclei is four [37]. In advanced gymnosperms such as *Welwitschia* and *Gnetum* (Gnetales)*,* there is no free nuclear stage [34].

In *P. ostii,* cell wall formation follows the coenocytic condition. Several meristematic centers are formed among peripheral cells of the proembryo. These centers produce various protuberances (embryonal tubes), each representing an independently developing embryo. In most cases, only one of the protuberances survives and matures. Batygina [38] proposed the term "embryoidogeny", which she defined as the formation of somatic embryos in the flower and seed and on vegetative organs in situ, in vivo and in vitro, as a new category of vegetative propagation. In this regard, the mature embryo of *P. ostii* and other *Paeonia* species [23, 31, 32] is a somatic embryo, arising from epidermal cells of the zygote embryo. The zygote embryo degenerates during seed development. Specifically, the zygote embryo (n + n) of *P. ostii* was observed before 45 DAP; thereafter, from 50 DAP a somatic embryo (embryoid, 2n → 2n) replaced the zygote embryo.

### Storage reserves

Accumulation of storage reserves such as proteins, carbohydrates and lipids is an important aspect of seed maturation. These reserves support germination and the initial growth of seedlings [39]. In *P. ostii,* the main species in the genus used for peony oil production, accumulation of storage reserves is similar to that of the oil seeds of *Arabidopsis thaliana* [40], *Brassica napus* [41], *Glycine max* [42] and *Sinapis alba* [43]. Specifically, at the beginning of seed development soluble sugar and starch content increased because carbohydrates produced by photosynthesis were translocated to seeds, thus promoting the accumulation of soluble sugar and starch [4, 39]. Subsequently, soluble sugars and starch are broken down, since they function as precursors in oil and protein synthesis [4, 39]. At the late stage of seed development, crude fat makes up the largest proportion of the reserves. Our results are consistent with those of Han et al. [15] who studied the effect of shading on nutrient accumulation of *P. ostii.* They found that soluble sugar and starch reached their maximum at 56 and 84 DAP, respectively, and that crude fat reached its maximum at 98 DAP. Zhao and Wu [44] also demonstrated that as seeds of *P. ostii* developed the number and size of the oil body increased. In *P. lactiflora* ‘Hangshao’ seeds, starch reached its maximum content at 60 DAP and crude fat at 75 DAP. Further, the crude fat content of *P. lactiflora* ‘Hangshao’ is lower than that of *P. ostii* [29].

Another point that needs to be mentioned is the coordinated interactions between the endosperm and seed coat during development of *P. ostii* seeds. Prior to 45 DAP, the principal tissue of the developing seed was the inner integument (seed coat), which contributed most of the seed mass. Therefore, during seed development of *P. ostii* the seed coat acts as a temporary storage tissue of the seeds, and the main storage reserves of seed coat are soluble sugars and starch. As the seed develops (i.e. as the endosperm is cellularized), thickness of seed coat decreases, while the endosperm contributes most of the seed volume (mass). At this time, lipid and protein become the main storage reserves. However, the interconversion of the storage reserves between endosperm and seed coat needs to be explored further.

### Germinability

Germinability of *P. ostii* seeds occurred 60–70 DAP, at which time 33% of them germinated. All seeds ≤ 60 DAP rotted (without germinating) during incubation. Germination percentage increased with seed development, indicating that the vigor of the embryos had increased. Furthermore, as seeds developed germination rate (speed) increased. Seeds reached maximum dry mass at 100 DAP, while germination percentage and rate were lower than they were at 110 and 120 DAP. Abscisic acid (ABA) induces seed maturation and promotes dormancy, while gibberellins antagonise ABA and promote germination [45]. During seed development in *P. ostii*, ABA content gradually increased and reached its maximum value at 104 DAP and then decreased [46]. Thus, we speculate this is why the physiologically matured seeds (100 DAP) had lower germination percentage and speed than seeds of 110 and 120 DAP. However, little is known about the content of GA during development of *P. ostii* seeds [46].

## Conclusions

**Fig. 9:**
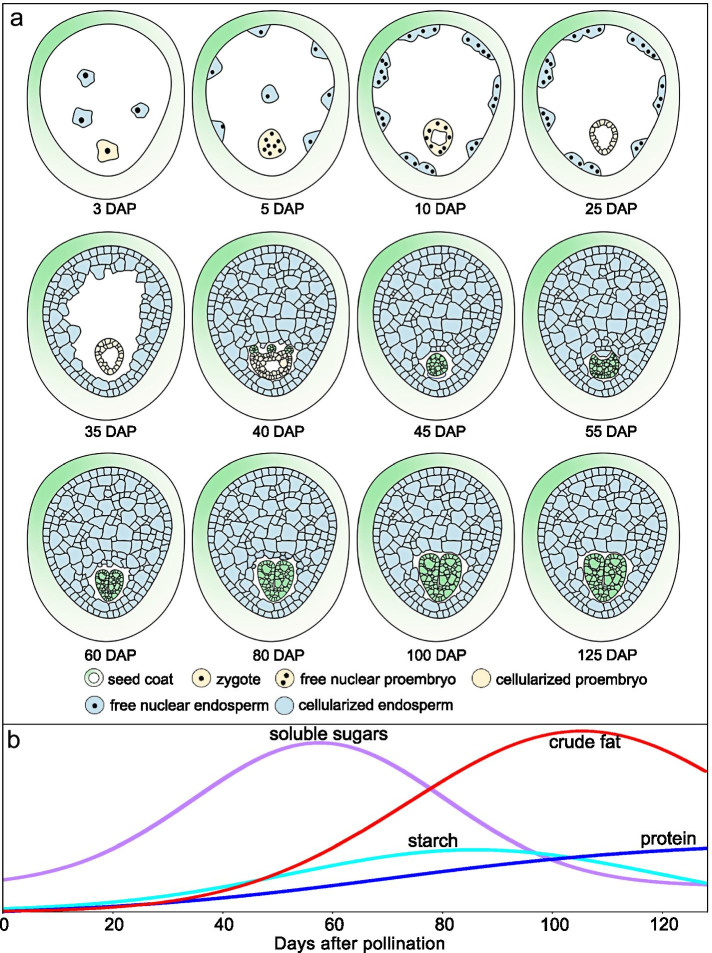
Summary of (a) seed development and (b) storage reserves in *Paeonia ostii*

The period of seed development in *P. ostii* from pollination to dispersal was 125 d, with seeds becoming germinable at 60–70 DAP and physiologically mature by 100 DAP. Like other orthodox seeds, those of *P. ostii* can be divided into early embryogenesis (phrase I), cell expansion and accumulation of stored reserves (phrase II) and maturation drying (phrase III). However, they differ from most other well-documented orthodox seeds, in which these three phrases are discrete and continuous. The first two phrases of *P. ostii* overlapped at 1–60 and 21–100 DAP. Loss of a large amount of water was not observed in phrase III. This possibly was due to accumulation of storage reserves in phrase II, which consumed a large amount of water, thus masking the water loss. Development of the embryo of *P. ostii* is similar to that reported for other peony species but differs from that which normally occurs in angiosperms. The development of *P. ostii* seeds and accumulation of storage reserves are summarized in Fig. 9. The ovule of *P. ostii* is crassinucellate and bitegmic. The inner integument is 2–4 cell layers thick and the outer integument 14–20 cell layers thick. The seed coat of *P. ostii* is derived from the outer integument. Endosperm development in *P. ostii* follows a typical nuclear type of development and is persistent. Embryo development can be divided into two stages: a coenocytic proembryo from zygote (n + n), and a somatic sexual embryo from peripheral cells of proembryo (embryoid, 2n → 2n). During seed development, the seed coat acts as a temporary storage tissue of the seeds. As the seed develops, the thickness of seed coat decreases. However, the interconversion of the storage reserves between endosperm and seed coat needs to be explored further.

## Methods

### Study site and seed collection

Plants of *P. ostii* used in this study were about 10-years old and were growing in a 0.3 ha experimental garden on the campus of Yangzhou University. Plants in this garden were owned by ourselves, which permit us unrestricted use. The plants used in this study were identified by Jun Tao from the Key Laboratory of Crop Genetics and Physiology of Jiangsu Province. To ensure seed set, flowers were hand cross-pollinated [47] in early April of 2018 and 2020. Then, every pollinated flower was tagged so that the pollination date was known. Fruits were collected on different days after pollination (DAP). The collected material was placed in ZipLoc plastic bags and taken to the laboratory immediately. Developing seeds used in all experiments were collected in 2018, except those used for determination of soluble sugars, starch, protein and crude fat content, which were collected in 2020.

### Seed morphology, fresh mass, dry mass and water content during development

Forty ovules/seeds (hereafter seeds) were separated from fruits collected at 5-day intervals between 0–125 DAP. Ten seeds (randomly-chosen from those 40 seeds) were used to measure seed length, width and thickness using a dissecting microscope and image analysis software for 0–35 DAP and a vernier caliper 40–125 DAP, after which seeds were photographed with a digital camera 40–125 DAP. Then, all 40 seeds were divided into four groups (i.e. four replicate of 10 seeds), and each group was weighed (fresh mass), oven-dried, weighed (dry mass) and water content calculated.

### Embryo morphology of developing seeds

To monitor morphological changes during seed development, 10 embryos were isolated from developing seeds harvested at 5-day intervals from 60–120 DAP using a razor blade. Then, embryo length and seed length were measured using a dissecting microscope and image analysis software. Embryo (E): seed (S) length ratios were calculated. The extremely small embryos at 0–55 DAP could not be seen by the naked eye.

### Germination of developing seeds

Four replicates of 25 seeds separated from fruits collected at 10-day intervals from 0–120 DAP were placed in 10-cm diameter Petri dishes on two layers of filter paper moistened with 5 mL distilled water and incubated at 15/25 °C in 12/12 h dark/light conditions. Cool white fluorescent tubes provided ca. 100 µmol/m^2^/s photon irradiance (400–700 nm) to seeds. Germination was checked daily for 125 days, and the criterion for germination was emergence of a radicle tip of ≥ 1 mm. Germinated seeds were counted and removed from the Petri dishes.

### Soluble sugars, starch, protein and crude fat content of developing seeds

The anthrone-H_2_SO_4_ colorimetric method was used to measure soluble sugar and starch content in the developing seeds [48]. Developing seeds were collected at 10-day intervals 20–120 DAP. After being ground and homogenized with 10 mL deionized water, 200 mg samples were put into a boiling water bath for 20 min. After being cooled to room temperature, the homogenate was centrifuged twice at 6000 rpm for 10 min at 15 °C. The supernatant was collected for determination of soluble sugar content at 630 nm via a UV spectrophotometer (Bluestar A, LabTech Ltd., Beijing, China). The residue was homogenized in 10 mL of deionized water and 2 mL of 52% perchloric acid for determination of starch spectrophotometrically at 630 nm [48].

Soluble protein content was assayed using the Coomassie brilliant blue G-250 method [48]. First, a Coomassie brilliant blue G-250 and standard protein solutions were prepared. One-half gram of fresh seeds was ground with 10 mL distilled water using a mortar and pestle. After centrifugation of the solution at 4000 × g for 10 min, the supernatant was transferred to a clean tube and total protein content measured as the change in absorbance at 595 nm [48].

Crude fat was determined by the Soxhlet extractor method [48]. One gram of developing seeds was ground and placed into a 50-mL Soxhlet extractor with an extraction time of 4 h in 100 mL n-hexane. Then, peony seed oil was extracted by distilling the n-hexane mixture in a rotary evaporator. The content of crude fat was calculated as described in [48].

### Anatomical changes in seeds during development

Ten developing seeds were collected at 2-day intervals from 0 (immediately before pollination) to 18 DAP and at 5-day intervals 20 and 110 DAP. The seeds were fixed in FAA (formalin: glacial acetic acid: 50% ethanol = 5: 5: 90 by volume) for 24 h. Then, they were dehydrated sequentially in 50%, 70%, 85%, 95% and 100% ethanol for 1 h in each concentration. Next, 100% ethanol was added to each sample for 1 h, after which samples were transferred sequentially to 25, 50, 75 and 100% tertiary-butanol for 4 h in each concentration. Then, each sample was embedded in paraffin wax and kept at ambient laboratory conditions until used. Finally, 6 µm longitudinal sections of the seed coat and pericarp were cut transversely using a microtome (RM2235, Leica Microsystems Inc., Heidelberg, Germany) and stained with 1% safranin followed by 2% fast green solutions. Observations and photography were carried out with a light microscope (Olympus CX31, Olympus Corp., Tokyo, Japan) and MShot 9.0 image analysis software (Micro-shot Corp., Shanghai, China).

### Statistical analysis

To evaluate the distribution pattern of seed length, width, thickness, water content, dry mass, fresh mass, soluble protein, soluble sugars, starch and crude fat during seed development, Linear, Gompertz, Hill, Logistic, Sigmoid, Gaussian and Weibull functions were used to fit the data in Sigmaplot ver. 12.0. The equation with the highest adjusted r^2^ was selected. Germination percentage data were normalized by arcsine-transformation and analyzed by one-way analysis of variance and Duncan's multiple range test in Statistica ver. 13 (Statsoft, Inc, Tulsa, OK, USA).

## Supplementary Information

**Additional file 1:** **Table S1.** Selected models fitted to seed length, width, thickness, dry mass, fresh mass, water content, starch, soluble protein, soluble sugar and crude fat.

## Data Availability

The data generated or analyzed in this study are included in this article and its supplementary information files. Other materials that support the findings of this study are available from the corresponding author on reasonable request.
