# Supplementary material for: Seed development in Paeonia ostii (Paeoniaceae), with particular reference to embryogeny
Source: BMC Plant Biol. 2021 Dec 18;21:603. doi: 10.1186/s12870-021-03373-z (PMC8684281; doi:10.1186/s12870-021-03373-z)
Supplement: Supplementary file 1 — Additional file 1: Table S1. Selected models fitted to seed length, width, thickness, dry mass, fresh mass, water content, starch, soluble protein, soluble sugar and crude fat. [file 12870_2021_3373_MOESM1_ESM.docx]

Supplementary data

Table S1 Selected models fitted to seed length, width, thickness, dry mass, fresh mass, water content, starch, soluble protein, soluble sugar and crude fat.

|  | Selected models | Equation | *r*^2^ |
| --- | --- | --- | --- |
| Seed length (0–100 DAP) | Sigmoid, 4 parameters | Y=-0.376+1.472/(1+exp(−(x–7.481)/17.348)) | 0.992 |
| Seed length (100–125 DAP) | Linear | Y=1.426–0.0034x | 0.863 |
| Seed width (0–100 DAP) | Sigmoid, 4 parameters | Y=-0.110+0.971/(1+exp(−(x–13.830)/12.979)) | 0.980 |
| Seed width (100–125 DAP) | Linear | Y=1.427–0.005x | 0.851 |
| Seed thickness (0–100 DAP) | Sigmoid, 4 parameters | Y=-0.036+0.78/(1+exp(−(x–18.63)/9.88)) | 0.990 |
| Seed width (0–100 DAP) | Linear | f=1.061–0.003x | 0.897 |
| Water content (100–125 DAP) | Sigmoid, 3 parameters | Y=89.671/(1+exp((x–103.779)/39.051)) | 0.989 |
| Dry mass | Sigmoid, 3 parameters | Y=0.310/(1+exp(−(x–58.678)/14.019)) | 0.990 |
| Fresh mass (0–100 DAP) | Hill, 3 parameters | Y=0.584×x^4.522^/(39.242^4.522^+x^4.522^) | 0.979 |
| Dry mass (100–125 DAP) | Linear | Y=0.993–0.004x | 0.918 |
| Starch | Gaussian, 3 parameters | Y=72.121×exp(−0.500×[((x–87.454)/34.128)^2^] | 0.923 |
| Protein | Sigmoid, 3 parameters | Y=39.612/(1+exp(−(x–38.192)/25.270)) | 0.927 |
| Soluble sugar | Gaussian, 4 parameters | Y=33.912+183.066×exp(−0.500×[((x–58.342)/23.282)^2^] | 0.955 |
| Crude fat | Gaussian, 3 parameters | Y=218.2572×exp(−0.500×[((x–103.586)/30.305)^2^] | 0.994 |
